# Enhancing the tumoricidal efficacy of the nanobubbles-mediated PD-L1 inhibition and immunogenic cell death in mice

**DOI:** 10.3389/fimmu.2025.1672324

**Published:** 2026-01-09

**Authors:** Yun Liu, Jiaxuan Han, Chaoqi Liu, Yezi Chen, Shiqi Yang, Yao Ma, Chang Zhou, Rong Liu, Yu Hu, Yun Zhao

**Affiliations:** 1 Department of Ultrasound Imaging, The First College of Clinical Medical Science, China Three Gorges University&Yichang Central People’s Hospital, Yichang, China; 2 Medical College of China Three Gorges University, Yichang, China; 3 Hubei Key Laboratory of Tumor Microenvironment and Immunotherapy, China Three Gorges University, Yichang, China; 4 Department of Ultrasound Imaging, Affiliated Renhe Hospital of China Three Gorges University, Yichang, China; 5 Department of Ultrasound Imaging, Shaanxi Provincial People’s Hospital, Shaanxi, China

**Keywords:** MiR-497, PD-L1, Shikonin, ICD, nanobubbles, anti-tumor immunotherapy

## Abstract

**Background:**

Immunotherapy becoming the focus of contemporary multidisciplinary collaborative research efforts towards advanced hepatocellular carcinoma (HCC).

**Objective:**

This study aims to develop a nanobubble (NB) delivery system designed to co-administer an immunogenic cell death (ICD) inducer, Shikonin (SK), alongside an immune checkpoint inhibitor, miR-497-5p, to enhance the efficacy of the immune response against liver cancer.

**Methods:**

NBs were synthesized through thin-film hydration and mechanical oscillation techniques to encapsulate miR-497 and SK. Comprehensive characterization and pharmacokinetic analyses of the miR-497/SK-loaded NBs were conducted both *in vitro* and *in vivo*. To evaluate the anti-tumor efficacy and immunological activity of this combination therapy, a subcutaneous transplanted tumor model using H22 hepatoma cells was established.

**Results:**

The miR-497/SK-NBs demonstrated an optimal morphology and size, as well as excellent gene capacity and SK encapsulation. The *in vivo* anti-tumor effects and mechanisms of SK and miR-497 were assessed in H22 hepatoma transplants model. The miR-497/SK-NBs group showed the strongest tumor inhibition, with their synergistic immunotherapeutic effect demonstrated through two main mechanisms. Initially, the activation of SK, facilitated by ultrasound, triggered the induction of injury-related molecular patterns, including CRT and HMGB1. This process subsequently activated CD80^+^ CD86^+^ macrophages, thereby enhancing the presentation of tumor antigens. Subsequently, miR-497 contributed to the downregulation of PD-L1 expression in tumor cells. Collectively, these processes elicited a robust systemic anti-tumor immune response and induced apoptosis in tumor cells within murine HCC models.

**Conclusions:**

The study demonstrated that miR-497/SK-loaded nanobubbles simultaneously boosts ICD and blocks the PD-1/PD-L1 pathway in immunotherapy. This finding offers a theoretical foundation for the effective eradication of tumor cells and the development of a highly efficient synergistic treatment strategy for HCC.

## 1 Background

The incidence of liver cancer has been on the rise globally, with significant increases noted in various regions, particularly in high socio-demographic index countries (1). Hepatocellular carcinoma (HCC), the predominant type of liver cancer, is linked to high mortality and poses significant treatment challenges (2). HCC treatments encompass liver transplantation, surgical resection, and systemic therapies such as targeted and immunotherapy. However, these treatments often have limitations, particularly in advanced stages of the HCC (3).

Tumor immunotherapy represents a groundbreaking cancer treatment strategy, focusing on immune checkpoints like programmed cell death protein 1 (PD-1) and its ligand, programmed death-ligand 1 (PD-L1), that suppress T cell activation and proliferation, enabling tumors to evade immune detection (4). At present, new strategy to target immune checkpoints are constantly being discovered, such as small RNAs (5, 6). MicroRNAs (miRNAs) are pivotal in HCC pathogenesis by modulating biological processes such as cell proliferation, invasion, and immune evasion (7). One specific miRNA, *miR-497a-5p*, has been shown to inhibit the expression of PD-L1 in tumor tissues (8). The downregulation of PD-L1 by *miR-497a* suggests a potential mechanism through which this miRNA may exert its tumor-suppressive effects in HCC. This interaction underscores the crucial role of miRNAs in regulating immune responses in the tumor microenvironment and suggests miR-497a can be a potential therapeutic target to boost anti-tumor immunity in HCC (9).

Sonodynamic therapy, as a non-invasive and highly targeted new strategy for tumor treatment, is demonstrating multi-dimensional therapeutic potential ranging from direct killing to immune regulation in the treatment of hepatocellular carcinoma. However, traditional sonodynamic therapy often faces limitations in the hypoxic microenvironment and the strong self-repairing ability of tumors. Recent studies have developed a new type of nano vesicle (ICG@C_3_F_8_-KL NBs) by combining disulfidptosis with sonodynamic therapy, achieving ultrasonic-responsive drug release and ROS generation, providing new ideas and methods for the treatment of HCC (10). Further, researchers have effectively alleviated tumor hypoxia by using *in situ* oxygenation of nano bubbles, not only significantly enhancing the efficiency of the sonodynamic effect, but also successfully promoting ferroptosis, a new type of cell death. At the same time, through RNA sequencing (RNA-seq) technology, the underlying molecular mechanism was deeply explored, confirming that sonodynamic therapy can specifically interfere with the antioxidant defense system of cells while triggering oxidative damage, jointly promoting the ferroptosis process (11). Beyond direct killing and induction of new cell death, the more profound significance of sonodynamic therapy lies in its ability to reprogram the immune microenvironment of liver cancer. Researchers have proposed a nano-sized ultrasound contrast agent (ICG@C3F8-R848 NBS) that can trigger macrophage polarization and immunogenic cell death (ICD) for the treatment of hepatocellular carcinoma (HCC) through sonodynamic therapy (SDT) and immunotherapy (12).

Immunogenic cell death (ICD) significantly stimulates the impaired antitumor immune system, particularly through adaptive immune responses mediated by cytotoxic T lymphocytes, and induces immunological memory when tumor cells undergoing death display adequate antigenicity (13). Recent research indicates that various novel modalities, such as Shikonin, can induce immunogenic cancer cell death (14, 15). Shikonin, a natural compound derived from the root of the lithospermum erythrorhizon plant, has garnered attention for its potential to induce ICD in cancer cells (16). This process is characterized by the release of damage-associated molecular patterns (DAMPs) that can stimulate an immune response against the tumor. Recent studies have demonstrated that shikonin not only triggers apoptosis but also necroptosis, thereby enhancing the immunogenicity of dying cancer cells (17). The ability of shikonin to induce ICD is particularly significant in the context of cancer therapy, as it can convert dying cancer cells into a form of vaccine that elicits a robust antitumor immune response. This is achieved through the exposure of immunostimulatory molecules, which are crucial for activating dendritic cells and promoting T cell responses (18). Furthermore, shikonin has been shown to modulate various signaling pathways associated with cell death, including the activation of the DNA damage response and the regulation of autophagy, which can further influence the immune landscape of the tumor microenvironment (19).

ICD and immune checkpoint have a close relationship in tumor immunotherapy. ICD can increase the immunogenicity of tumor cells, release DAMPs, activate adaptive immune responses, and immune checkpoint inhibitors can enhance these immune responses, restoring T cell recognition and killing ability towards tumor cells (20). Therefore, the combined use of ICD and immune checkpoint inhibitors may become an effective tumor treatment strategy, improving treatment efficacy by activating and enhancing the body’s anti-tumor immune response (21).

In this experiment, we designed a combination of ICD inducer (Shikonin) and immune checkpoint inhibitor (*miR-497-5p*) to enhance anti-tumor immune response, while using ultrasound microbubbles as carriers, which is beneficial for targeted drug release, especially for protecting drug degradation (22). The ultrasound-induced cavitation effect and mechanical vibrations facilitated the disruption of NBs, thereby enhancing cellular uptake of the delivered drugs and genes. Subsequently, the synergistic anti-tumor efficacy of ultrasound-mediated miR-497/SK-NBs on HCC was assessed. The investigation included the evaluation of tumor apoptosis, immunogenic cell death, and the activation of the immune system.

## 2 Methods

### 2.1 Cell cultures and animal models

Hepa1–6 cells and H22 were purchased from China Center for Type Culture Collection (CCTCC). Hepa1–6 cells were maintained in high glucose DMEM medium, while H22 cells were cultured in 1640 medium, both supplemented with 10% FBS and 100 units/ml penicillin G/streptomycin (Thermo Fisher, Waltham, MA, USA).

Male BALB/c mice, weighing between 18 and 22 g, were obtained from China Three Gorges University, with ethical clearance secured from the Medical Animal Care & Welfare Committee of China Three Gorges University (Ethical approval number: 2022050). And the research have complied with the ARRIVE guidelines.

### 2.2 Preparation of miR-497/SK-NBs

The miR-497-targeted genes were predicted utilizing the TargetScan online database (https://www.targetscan.org/). A total of 372 HCC patients data generated by the TCGA Research Network (https://www.cancer.gov/tcga). The expression levels of has-miR-497-5p were compared between 372 cases of HCC tissue and 50 cases of adjacent non-cancerous tissue. This study utilized publicly available, anonymized datasets from TargetScan and TCGA. All data have been thoroughly de-identified in accordance with HIPAA privacy standards.

The preparation of cationic nanobubbles used the method of thin-film hydration. miR-497a-5p/SK-NBs consisted of four components: DPPC, DSPE-PEG2000, Dc-cholesterol and SK with a mass ratio equivalent to 5:2:0.5:0.5, respectively. The lipid mixture was initially dissolved in chloroform within a round-bottom flask. The organic solvent was removed by rotary evaporation (Precision HLG3; Heidolph), and a thin film of phospholipids was formed on the walls of the round-bottomed flask.

A prewarmed glycerin PBS mixture (Glycerin: PBS = 1:9) at 37°C was added to the dried lipids and incubated in a 45°C water bath until complete dissolution. The liposome suspension were stored at 4°C in penicillin bottles. Perfluoropropane gas (Wuhan Newradar Special Gas Co., Ltd., Wuhan, China) was introduced into the vials using a custom gas exchange device. The gaseous mixture was agitated for 120 seconds using a High-speed oscillator (SI Vortex-Genie 2; Scientific Industries; Thermo Fisher Scientific, Inc.). A miRNA-497 plasmid was combined with cationic lipid microbubbles at a 1:25 ratio and incubated on a flat rocker for 60 minutes to promote miRNA-nanobubble interaction.

### 2.3 Characterization of miR-497/SK-NBs

The NBs were dried and coated at the critical point, then examined using SEM (JSM-7500F, JEOL, Tokyo, Japan) to capture images of their form and size for further analysis. Vesicle size was determined using a Nano-ZS Zetasizer (Malvern Instruments, UK).

The encapsulation efficiencies (EE) of SK-NBs and miR-497/SK-NBs were determined by dialysis. Initially, the absorbance (Optical Density, OD) of SK at varying concentrations was determined using an ultraviolet spectrophotometer. Subsequently, a standard curve was established based on the correlation between SK concentration and OD values. A dialysis bag (Union Carbide, US) was employed to dialyze SK-NBs (miR-497/SK-NBs) for six hours to remove free SK.The sample was then mixed with methanol to dissolve the NBs and release the encapsulated SK. The SK encapsulated within the NBs was isolated, and its OD value was measured using an ultraviolet spectrophotometer. The concentration of SK was then calculated using the previously established standard curve. Entrapment rates were calculated using the following equation: EE% = (1-Weight _free drug_/Weight _total drug_) × 100%.

To determine the maximum gene carrying capacity of NBs, different concentrations were mixed with 1 µg of miRNA plasmid and incubated at 4°C for 1 hour to ensure full binding of miR-497 to the NBs. Subsequently, gel electrophoresis was employed to evaluate the interaction between NBs and plasmid, thereby determining the optimal loading capacity.

### 2.4 Luciferase reporter

A luciferase reporter assay was conducted using the pMIR-REPORT vector to validate the interaction between miR-497 and PD-L1 by evaluating PD-L1 expression levels after co-transfecting miR-497 with the reporter gene.Hepa1–6 cells were initially co-transfected with a luciferase reporter gene plasmid and either miR-497a-5p mimic, its negative control (NC), miR-497a-5p inhibitor, or its inhibitor NC.Each experimental condition was replicated in triplicate.After 48 hours, the luciferase activity detection kit (Vazyme Biotech Co., China) was used to prepare the working solution as per the manufacturer’s instructions, which was then added to the cultured cells. Following cell lysis, fluorescent substrates were introduced, and fluorescence intensity were measured using an automated enzyme marker (Shanghai Precision Instrument Co., LTD.) to assess luciferase activity.

### 2.5 Animal model establishment

Fifty female SPF BALB/c mice, each weighing between 18 and 20 g and aged 6 to 8 weeks, were sourced from the Animal Experimental Center of China Three Gorges University. To establish a subcutaneous mouse hepatocarcinoma model, H22 cells, at a concentration of 1×10^6^ cells per 100 µL and in the logarithmic growth phase, were subcutaneously injected into the right anterior axillary region of the mice. The growth of the subcutaneously transplanted tumors was monitored every two days. Pharmacological treatment began once the average tumor diameter measured between 5 mm and 7 mm. All animal experiments adhered to the National Institute of Health’s Guidelines for the Care and Use of Laboratory Animals and were approved by the Laboratory Animal Management Committee at China Three Gorges University.

Forty-two mice with tumor diameters of 5–7 mm were randomly divided into five groups, each containing seven mice (n=7). Including Contol group, NBs group, SK group, miR-497-NBs group, SK-NBs group and miR-497/SK-NBs group. NBs were injected into the tail vein, with each dose containing 1×10^11^ NBs in 200 µL. The control group received an injection of 200 µL of normal saline. Subsequently, the tumor was subjected to ultrasound irradiation for 120 s following each injection. The irradiation parameters were set as follows: power density of 1 W/cm², frequency of 1 MHz, and a duty cycle of 50%, for a total of five treatments, alternate days of treatment were used. Before each treatment, mice were weighed, and tumor dimensions, including the long diameter (a) and short diameter (b), were measured with vernier calipers. The tumor growth curve was generated by determining the tumor volume at various time intervals using the formula (1/2ab^2^). Subsequently, the tumor volume inhibition rate was calculated.

To evaluate the potential systemic toxicity of NBs, body weights and changes in vital organs were documented at the end of the treatment period. Additionally, hepatic function markers were assessed through post-treatment blood sample analysis. Including serum levels of alanine aminotransferase (ALT), aspartate aminotransferase (AST), and alkaline phosphatase (ALP) were quantified.

To evaluate the effects of different drug groups on tumor tissues and organs, specimens were dehydrated, embedded in paraffin, and sectioned into 4 µm slices. The sections were stained with hematoxylin and eosin (H&E) to observe morphological changes in tumor tissues and organs under the optical microscope. Tumor tissue sections were stained with the TUNEL apoptosis detection kit (Meilunbio, China) to assess cellular apoptosis, and observations were made using an inverted fluorescence microscope.

### 2.6 Immunohistochemistry

To examine apoptosis in tumor tissues post-drug treatment, tissue sections were dewaxed, hydrated, and underwent antigen retrieval. The sections were treated with 3% hydrogen peroxide for 30 minutes to remove endogenous peroxidase activity.To minimize non-specific protein binding, the samples were blocked with 5% fetal bovine serum albumin (BSA, Solarbio, China) for 1 hour, followed by overnight incubation with Bax and Bcl-2 antibodies, each at a 1:200 dilution (Santa Cruz Biotechnology, USA). Subsequently, the slices were incubated with the appropriate secondary antibody at room temperature for one hour.Following incubation, the slices were washed three times with PBS. Following color development with 3,3’-diaminobenzidine (DAB, Biological Technology Co. Ltd.), the slices underwent hematoxylin counterstaining. After an additional wash, the slides were sealed with neutral resin adhesive post-staining. After drying, images were taken with an inverted fluorescence microscope and analyzed semi-quantitatively using Image J software.

### 2.7 Immunofluorescence

Tumor sections were incubated with primary antibodies (PD-L1, CRT, IFN-γ [Proteintech Co. Ltd., China], CD8a, CD80, and CD86 [BioLegend Co. Ltd., USA]) in a humidified chamber at 4°C for 12–16 hours, followed by three PBS rinses. Subsequently, they were incubated with a fluorescently labeled secondary antibody for 1 hour and then washed again with PBS. And the sections underwent DAPI counterstaining for cell nuclei. Fluorescence microscopy was employed to visualize the expression of target proteins in cells or tumor tissues.Semi-quantitative analysis of the target proteins was conducted using ImageJ software.

### 2.8 Reverse transcription-quantitative PCR

RT-qPCR was used to quantify gene expression. RNA was extracted from tumor tissues of each experimental group using the Trizol method and stored at -80°C. The isolated RNA was reverse transcribed into complementary DNA (cDNA) using a reverse transcription kit from Vazyme Biotech Co., China. The synthesized cDNA was mixed with specific primers for genes such as Bax, Bcl-2, TNF-α, IFN-γ, PD-L1, CD8, CD80, and CD86, along with SYBR qPCR Master Mix (Vazyme Biotech Co., China) for qPCR analysis. Each cDNA sample was analyzed using the Applied Biosystems 7900HT Fast Real-Time PCR System (Applied Biosystems, Foster City, CA, USA). The thermal cycling protocol included an initial pre-denaturation at 95°C for 20 seconds, followed by a denaturation phase at 95°C for 3 seconds and an annealing phase at 60°C for 30 seconds. An extension phase at 72°C for 1 minute was conducted, repeated over 40 cycles.The 2-ΔΔCt method was used to calculate the relative gene expression levels, with β-actin as the internal control. The primer sequences are detailed in **Table 1**.(5’ to 3’).

**Table 1:** The sequences of primers.

Gene	Forward	Reverse
GAPDH	5’-GGTGGTCTCCTGTGACTTCAA-3’	5’-CCACCCTGTTGCTGTAGCC-3’
Bax	5’-TGGTTGCCCTCTTCTACTTTGC-3’	5’-CAGACAAGCAGCCGCTCAC-3’
Bcl-2	5’-CGTCAACAGGGAGATGTCACC-3’	5’-CAGCCAGGAGAAATCAAACAGAG-3’
HMGB1	5’-GCATCCTGGCTTATCCATTGG-3’	5’-GCTTTCTTCTCATAGGGCTGCTTG-3’
CD80	5’-TCTCCACGGAAACAGCATCT-3’	5’-CTTACGGAAGCACCCATGAT-3’
CD86	5’-GGCAAGGCAGCAATACCTTA-3’	5’-CTCTTTGTGCTGCTGATTCG-3’
PD-L1	5’-TGCTGCATAATCAGCTACGG-3’	5’-CCACGGAAATTCTCTGGTTG-3’
INF-γ	5’-CCATCGGCTGACCTAGAGAA-3’	5’-GATGCAGTGTGTAGCGTTCA-3’
TGF-β	5’-TGACGTCACTGGAGTTGTACGG-3’	5’-GGTTCATGTCATGGATGGTGC-3’
IL-2	5’-ACTTGGACCTCTGCGGCA-3’	5’-CCACCACAGTTGCTGACTCATC-3’

### 2.9 Flow cytometry

PD-L1 expression detection: The down-regulation of PD-L1 by miR-497a-5p was assessed using flow cytometry. Initially, cultured Hepa1–6 cells were transfected with a miR-495-5p mimic, a miR-497a-5p mimic negative control (NC), a miR-497a-5p inhibitor, or a miR-497a-5p inhibitor NC. Each experimental group included three replicate wells. Hepa1–6 cells were stained with an APC-PD-L1 antibody and incubated in the dark for 30 minutes after 48 hours. Post-centrifugation, flow cytometry was employed to quantify PD-L1 expression levels on Hepa1–6 cell surfaces across all groups.

To assess the cell apoptosis induced by various drugs in Hepa1–6 cells, cells exhibiting stable proliferation were exposed to different drugs for 24 hours. Subsequently, the cells were double-stained with an apoptosis detection kit (Vazyme Biotech Co., China), incubated in the dark for 30 minutes, then centrifuged and washed three times. The apoptotic proportion of Hepa1–6 cells in each experimental group was quantified via flow cytometry.

To detect CRT expression, Hepa1–6 cells were treated with drugs as previously described, stained with a FITC-CRT antibody, incubated in the dark for 30 minutes, then centrifuged and washed three times. Flow cytometry was used to measure CRT expression on the cell surface.

To detect lymphocyte proliferation, Spleens were harvested from each group, and spleen cell suspensions were prepared by grinding. Each group was divided into three control subgroups. Cell suspensions were incubated with CFDA-SE (Meilun Bio, China) for 30 minutes before being transferred to 24-well plates.Each well received tumor antigen and interleukin-2, followed by incubation at 37°C with 5% CO_2_ for 48 hours. Spleen cells were harvested, washed thrice with PBS, centrifuged at 1500 rpm for 5 minutes, and analyzed for splenic lymphocyte proliferation via flow cytometry.

To detect NK and macrophage cells, the homogenized splenic cell suspension was incubated with an appropriate volume of diluted PE-labeled anti-mouse NK1.1 antibody (Biolegend, USA) in the dark for 30 minutes. After three centrifugation and washing cycles, flow cytometry was used to evaluate the NK cell proportion in the splenic cell suspension for each group.Simultaneously, the diluted FITC - CD80 and APC - CD86 antibodies (Biolegend, USA) were introduced into the homogenized splenic cell suspension.This mixture was incubated in darkness for 30 minutes and subsequently subjected to three rounds of centrifugation. Flow cytometry was used to quantify the macrophage proportion in the splenic cell suspension for each experimental group.

### 2.10 Enzyme linked immunosorbent assay

To evaluate the expression of HMGB1 in tumor tissues across various treatment groups, an ELISA assay was employed to quantify HMGB1 and ATP levels. The standard provided in the Novozan Bio ELISA kit was serially diluted to construct a standard curve. Subsequently, 50 µL of the standard served as the control group. A 10 µL sample was mixed with 40 µL of diluent and incubated at 37°C for 30 minutes. The sample was diluted 30-fold and 50 µL of enzymatic reagent was added. Subsequently, 50 µL each of color developing agents A and B were added to each well. The reaction mixture was incubated at 37°C for 10 minutes, shielded from light. The optical density (OD) of each well was measured at 450 nm using an ELISA reader.

### 2.11 Statistical analysis

The experimental data were statistically analyzed using SPSS 18.0 (SPSS Inc., Chicago, IL, USA).Data are expressed as mean ± standard deviation. A t-test was used to determine significant differences between groups, and one-way analysis of variance (ANOVA) was applied for assessing differences among multiple groups. A p-value of less than 0.05 was considered indicative of statistical significance. A p-value of less than 0.01 or 0.001 were regarded as extremely significant.

## 3 Results

### 3.1 Bioinformatics analysis and verification of a *miR-495a-5p* target gene *CD274*

**Figure 1:**
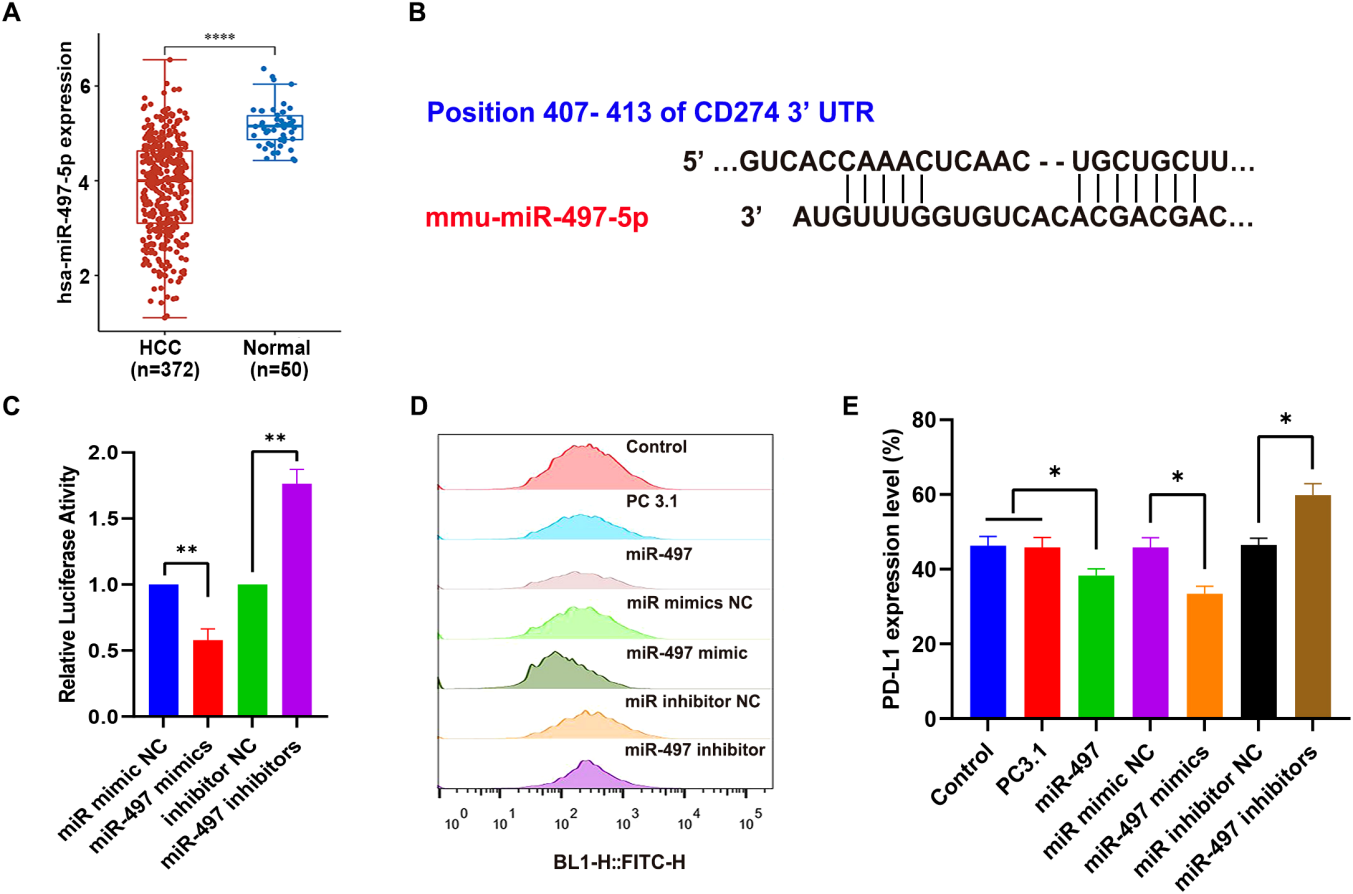
Effect of *miR-497* on the expression of PD-L1 *in vitro*. **(A)** The expression levels of has-miR-497-5p were compared between HCC cancerous tissues and adjacent non-cancerous tissues (^****^p<0.0001). **(B)** The sequences of miR-497 and its potential binding sites within the 3’UTR of PD-L1 are predicted. **(C)** The relative luciferase activity was assessed for the activity of the miR-497 targeting CD274 (PD-L1) using luciferase reporter gene assay (**P< 0.01). **(D)** Flow cytometry was utilized to detect PD-L1 expression in Hepa 1–6 following transfection with miR-497, as well as its mimic or inhibitor. **(E)** Quantitative analysis of **(D)** to evaluate PD-L1 expression in Hepa 1–6 cells post-transfection with miR-497, its mimic, or inhibitor (^*^P< 0.05). PC3.1 (pcDNA3.1); miR-497 (pcDNA3.1-pri-miR-497).

Initially, a total of 372 HCC mass and 50 paracancerous tissue were retrieved from the Cancer Genome Atlas (TCGA) database to examine the expression levels of hsa-miR-497-5p (**Figure 1A**). The analysis revealed a significant downregulation of hsa-miR-497-5p expression in HCC. Subsequently, bioinformatics tools were employed to investigate the potential target-effect relationship and binding sites between miR-497 and the PD-L1 gene (**Figure 1B**). The findings indicated the presence of effective binding sites, suggesting a potential target-effect relationship between miR-497 and PD-L1.

The study involved the construction of a pcDNA3.1-pri-miR-497 plasmid and a targeted PD-L1 3’-UTR luciferase reporter gene plasmid, alongside the synthesis of miR-mimic NC, miR-497 mimics, miR-inhibitor NC, and miR-497 inhibitors. The constructs were co-transfected into Hepa1–6 cells, followed by the measurement of relative fluorescence units (RFU) (**Figure 1C**). The regulatory interaction between the miR-497 gene and PD-L1 was subsequently analyzed. Furthermore, miR-497 was transfected into Hepa1–6 cells to assess its impact on PD-L1 expression levels (**Figures 1D, E**). The results suggest that miR-497 effectively targets PD-L1, thereby modulating its expression.

### 3.2 Preparation and characterization of miR-497/SK-NBs

**Figure 2:**
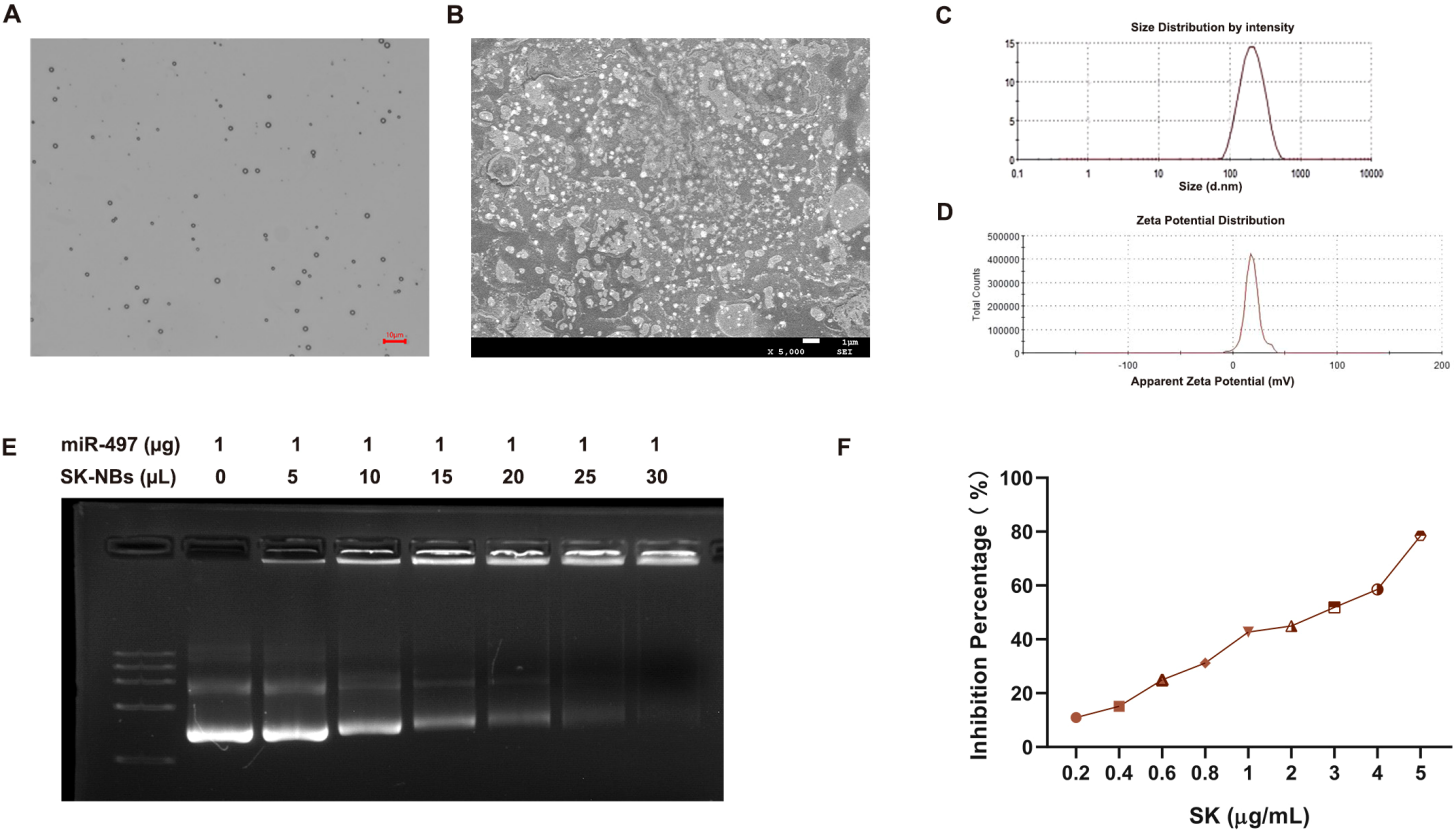
Characterization of miR-497/SK-NBs. **(A)** Confocal microscopy of miR-497/SK-NBs (magnification, 600×), scale bar 10 μm. **(B)** Scanning electron microscopy of miR-497/SK-NBs (magnification, 5000×), scale bar 1 μm. **(C)** Particle size of miR-497/SK-NBs. **(D)** Zeta potential of PD-L1 miR-497/SK-NBs. **(E)** Gel electrophoresis analysis demonstrating the miRNA loading capacity of miR-497/SK-NBs. **(F)** The cytotoxic effect of SK on Hepa 1–6 cells was assessed using the MTT assay.

The NBs were synthesized using the thin-film hydration method. SK-NBs and miR-497/SK-NBs form a lilac suspension. We utilized optical microscopy and transmission electron microscopy (TEM) to examine the morphology of miR-497/SK-NBs (**Figures 2A, B**). The miR-497/SK-NBs showed nearly spherical, with uniform size and distribution. The particle size of miR-497/SK-NBs, as determined by a particle size analyzer, was approximately 358 ± 55 nm (**Table 2**, **Figure 2C**). NBs incorporating Dc-chol can exhibit positive charges, facilitating their binding to miRNA through electrostatic adsorption (**Figure 2D**). Agarose gel electrophoresis images indicated that the NBs exhibited a maximum miRNA binding capacity of 1 µg/25 µL (**Figure 2E**). The cytotoxic effects of SK on Hepa 1–6 cells were assessed using the MTT assay. An incremental increase in SK concentration corresponded to a progressive enhancement in cytotoxicity towards Hepa 1–6 cells. The concentration of SK at 3 μg/mL was found to approximate the half-maximal inhibitory concentration (IC50) (**Figure 2F**). Consequently, this concentration was utilized in subsequent cellular experiments. The encapsulation efficiency of SK-NBs and miR-497/SK-NBs, assessed through thin film dialysis, was 47.68 ± 5.59% and 42.57 ± 4.55%, respectively.

**Table 2:** The characteristics of nanobubbles.

Group	The average diameter (nm)	Zeta potential (mV)	PDI	Encapsulation efficiency of SK (%)
NBs	307 ± 32	10.8 ± 3.7	0.163 ± 0.035	/
miR497-NBs	287 ± 44	7.2 ± 4.4	0.209 ± 0.027	/
SK-NBs	329 ± 41	9.1 ± 3.6	0.175 ± 0.051	47.68 ± 5.59
miR-497/SK-NBs	358 ± 55	6.9 ± 2.4	0.195 ± 0.047	42.57 ± 4.55

(Data are expressed as mean ± SD (n = 3). Statistical significances were calculated via t-Test).

### 3.3 *In vivo* antitumor and safety evaluation

**Figure 3:**
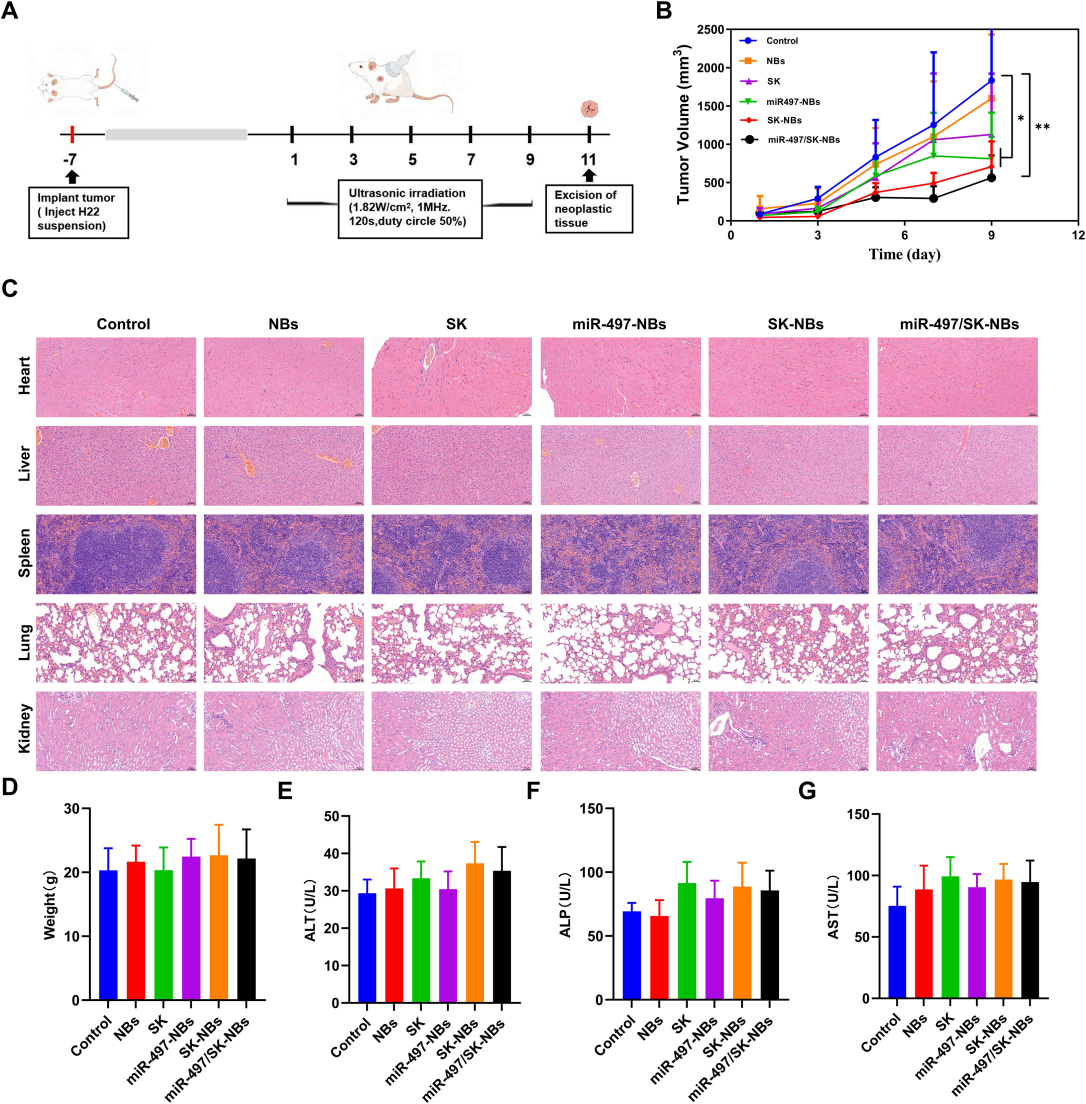
The inhibitory impact of miR-497/SK-NBs on subcutaneously transplanted hepatocellular carcinoma tumors in mice. **(A)** Schematic representation of the therapeutic formulations administered *in vivo*. **(B)** Tumor growth curves following various treatment protocols. **(C)** HE staining results of important organs in each group of mice at the endpoint of treatment, (magnification, 200×), scale bar 50 μm. **(D)** Comparison of body weights of mice in each group at the treatment endpoint. Comparison of liver function and biochemical indexes such as ALT **(E)**, ALP **(F)** and AST **(G)** of mice in each group at the endpoint of different treatments. (Data are expressed as mean ± SD (n = 5). Statistical significances were calculated via ANOVA). *P < 0.05, **P < 0.01 versus the control group.

We investigated the synergistic antitumor effects of miR-497 and SK in conjunction with the NBs platform in H22 tumor-bearing mice. This is illustrated in the schematic diagram in **Figure 3A**. The tumor growth curve and volume (weight) inhibition rate are presented in **Figure 3B**, **Table 3**, respectively. The findings consistently demonstrated a similar trend of tumor inhibition. Tumor growth in the control group was rapid, whereas treatments with free SK, miR-497-NBs, SK-NBs, and miR-497/SK-NBs resulted in varying degrees of tumor growth delay. Treatment with free SK only marginally reduced tumor growth, likely due to its nonspecific targeting of the tumor. However, when SK was encapsulated within the NBs, the resulting SK-NBs significantly decreased tumor volume and weight inhibition rates (61.32% and 59.73%, respectively; *p<0.05*). Significantly, the miR-497/SK-NBs group demonstrated a pronounced therapeutic effect. The tumor volume and mass in the miR-497/SK-NBs group (562.52 ± 290.69 mm³, 1.07 ± 0.46 g) were reduced by approximately one-third compared to the control group (1632.27 ± 420.76 mm³, 2.93 ± 1.62 g). Additionally, the inhibitory rates of tumor volume and weight for miR-497/SK-NBs were 65.54% and 63.48%, respectively (*p<0.01*), indicating an enhancement of antitumor efficacy through the combination of SK and miR-497. While the quantitative data on tumor volume and weight demonstrated a significant treatment effect, we acknowledge that this study has a limitation in the absence of representative photographic records of the excised tumors. .

**Table 3:** Comparison tumor growth inhibition in different groups.

Group	Tumor volume (mm^3^)	Volume inhibition rate (%)	Tumor weight (g)	Weight inhibition rate (%)
Control	1632.27 ± 420.76	0	2.93 ± 1.62	0
NBs	1599.43 ± 834.85	2.01	2.65 ± 1.24	9.56
SK	1126.89 ± 794.12	30.96	2.04 ± 0.97	30.38
miR497-NBs	809.76 ± 602.65^*^	50.39^*^	1.62 ± 0.62	44.71
SK-NBs	708.61 ± 327.98^*^	56.59^*^	1.18 ± 0.59	59.73
miR-497/SK-NBs	562.52 ± 290.69^**^	65.54^**^	1.07 ± 0.46^*^	63.48^*^

(Data are expressed as mean ± SD (n = 5). Statistical significances were calculated via ANOVA. Compared with the control group, *P < 0.05, **P < 0.01).

Heart, liver, spleen, lungs and kidneys of mice in each group were taken for HE staining to assess the safety of the treatment means in each group (**Figure 3C**). Compared with the control mice, the remaining groups of mice did not show any significant abnormal organic changes in the vital organs. There was no significant difference in the body weight of mice in each treatment group compared to the control group at the end of treatment (**Figure 3D**). In order to further compare the safety of the above treatments, blood was taken from each group of mice to evaluate the liver function indexes of ALT, AST and ALP, and found that the liver function indexes of the mice in the miR-497/SK-NBs group were basically at the same level as that of the control group (**Figures 3E-G**).

### 3.4 The miR-497/SK-NBs mediated apoptosis in cancer cells

**Figure 4:**
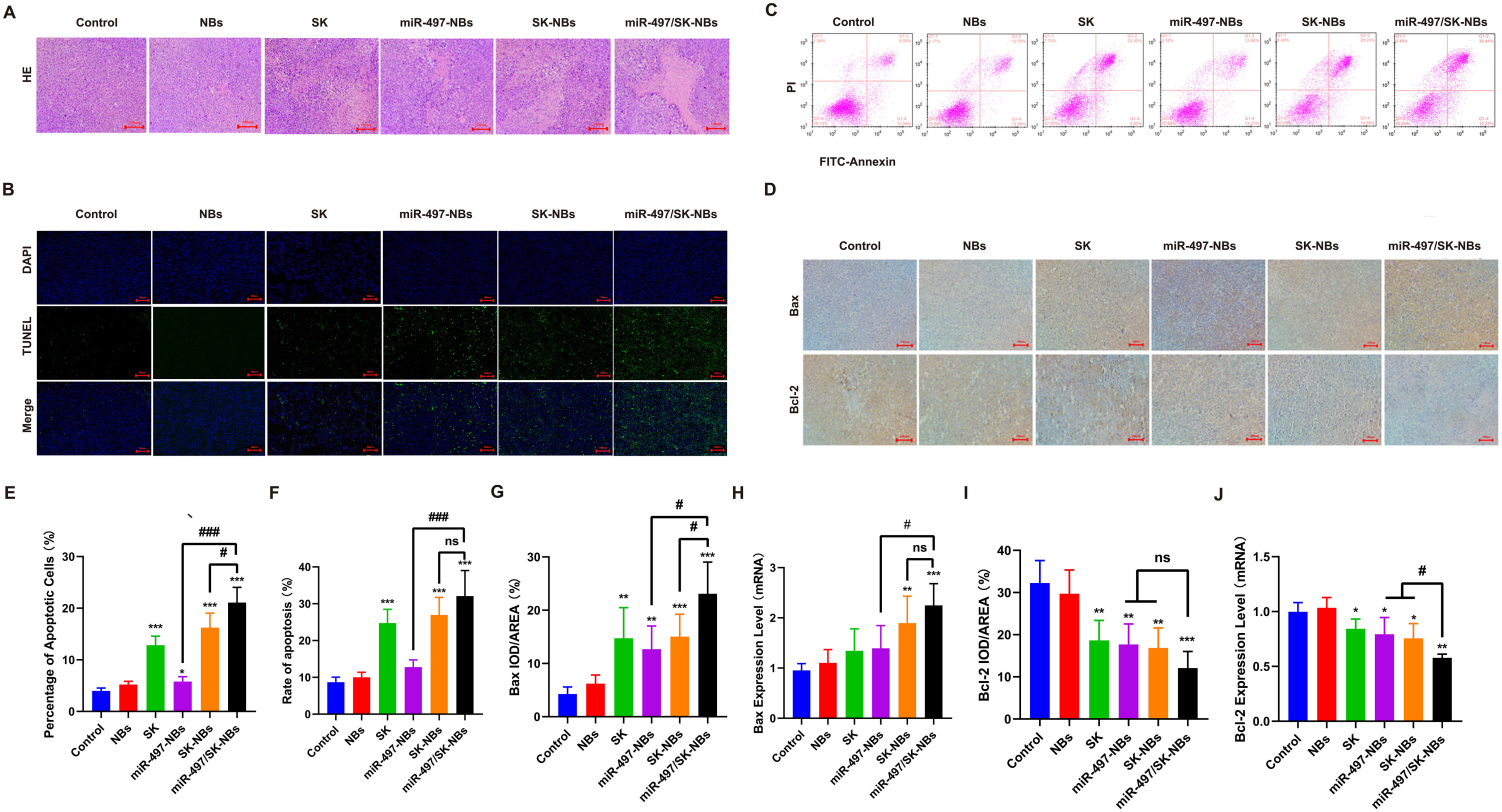
Analysis of apoptosis in tumor tissues. **(A)** Histological examination by HE staining (magnification, 200×), scale bar 100 μm. **(B)** TUNEL images of tumor tissues (magnification, 200×), scale bar 100 μm. **(C)** Apoptosis rates were assessed by flow cytometry. **(D)** Immunochemistry staining images of Bax and Bcl-2 (magnification, 200×), scale bar 100 μm. **(E)** Semi-quantification of the fluorescence intensity shown in panel **(B)**. **(F)** A column analysis of **(C)** in tumor tissues apoptosis rates. **(G, H)** Semi-quantification of Bax protein and mRNA. **(I, J)** Semi-quantification of Bcl-2 protein and mRNA. (Data are expressed as mean ± SD (n = 5). Statistical significances were calculated via ANOVA. ^*^P < 0.05, ^**^P < 0.01, ^***^P < 0.001 versus the control group, ^#^P < 0.05, ^###^P < 0.001 versus the miR-497/SK-NBs group).

To elucidate the underlying mechanisms by which miR-497/SK-NBs enhance apoptosis in cancer cells, we conducted histopathological and apoptosis analyses using H&E staining, TUNEL assay, and flow cytometry in tumor tissues. H&E staining revealed degeneration and necrosis in all treatment groups, with the miR-497/SK-NBs group exhibiting the most significant effects (**Figure 4A**). The control group exhibited active cell proliferation, characterized by large, hyperchromatic nuclei, an increased nucleocytoplasmic ratio, and marked atypia. The miR-497/SK-NBs group exhibited an approximately fourfold increase in apoptotic cell proportion compared to the control group (*P < 0.0001*) (**Figures 4A-F**). These findings suggest that the miR-497/SK-NBs group experienced significant histological damage and a high degree of apoptosis, contributing to the inhibition of tumor growth.

Furthermore, tumor cell apoptosis was assessed by quantifying apoptosis markers, specifically Bax and Bcl-2, within the tumor mass of mice administered various formulations (**Figure 4D**). The study demonstrated a notable increase in Bax protein expression in both the SK and miR-497 groups relative to the control group (*P < 0.001*). Furthermore, the miR-497/SK-NBs group exhibited a significantly higher expression than the SK-NBs and miR-497-NBs groups (*P < 0.0*5) (**Figure 4G**). Conversely, Bcl-2 protein expression exhibited an inverse pattern across all groups (**Figure 4I**), thereby facilitating the apoptotic process.RT-qPCR analysis confirmed that the gene expression levels of Bax and Bcl-2 aligned with the observed protein expression patterns.4H&J). The decrease in Bcl-2 and increase in Bax confirmed apoptosis in cancer cells, indicating that miR-497/SK-NBs treatment may boost CTL-mediated cytotoxicity through the mitochondrial apoptosis pathway.

### 3.5 The miR-497/SK-NBs promoted tumor cell immunogenic cell death and macrophage activation

**Figure 5:**
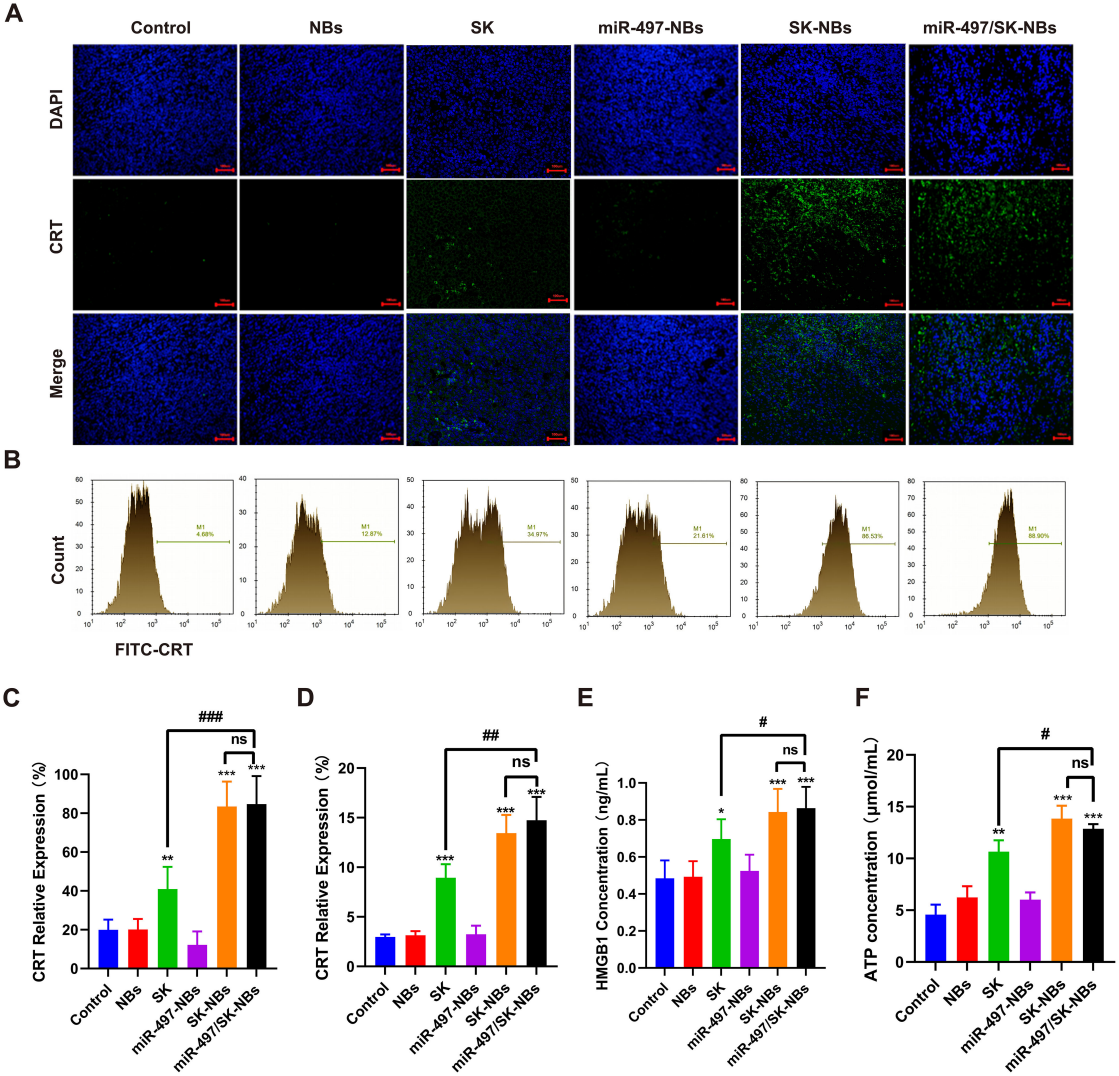
Investigate the regulatory effects of miR-497/SK-NBs on ICD markers within tumor tissues. **(A)** CRT expression in tumor tissues was assessed using immunofluorescence microscopy (magnification, 200×), scale bar 100 μm. **(B)** CRT expression on the cell surface was evaluated via flow cytometry. **(C)** Quantitative analysis of immunofluorescence data from panel **(A)**. **(D)** Quantitative analysis of CRT expression from panel **(B)**. The secretion level of HMGB1 **(E)** and ATP **(F)** in tumor tissues was measured using ELISA. (Data are expressed as mean ± SD (n = 5). Statistical significances were calculated via ANOVA. ^*^P < 0.05, ^**^P < 0.01, ^***^P < 0.001 versus the control group, ^#^P < 0.05, ^##^P < 0.01, ^###^P < 0.001 versus the miR-497/SK-NBs group).

ICD is a form of cell death that stimulates the immune system to recognize and attack tumor cells. This process is characterized by the release of DAMPs that activate antigen-presenting cells, such as macrophages, leading to an enhanced immune response. To verify the effect of miR-497/SK-NBs on ICD at the tumor site, we performed immunofluorescent (**Figure 5A**), flow cytometer (**Figure 5B**) and ELISA (**Figures 5E, F**) to evaluate the density of CRT and HMGB1 in tumor masses. We observed the significant improvements were demonstrated by favorable increases of the HMGB1 and CRT levels in the tumor masses recovered from contain SK groups according to the following order, miR-497/SK-NBs > SK-NBs > free SK groups, as compared to those levels in the control tumor masses (**Figures 5C-F**). This observation suggests that the SK had the potential to induce substantial immunogenic cell death in tumor tissues.

**Figure 6:**
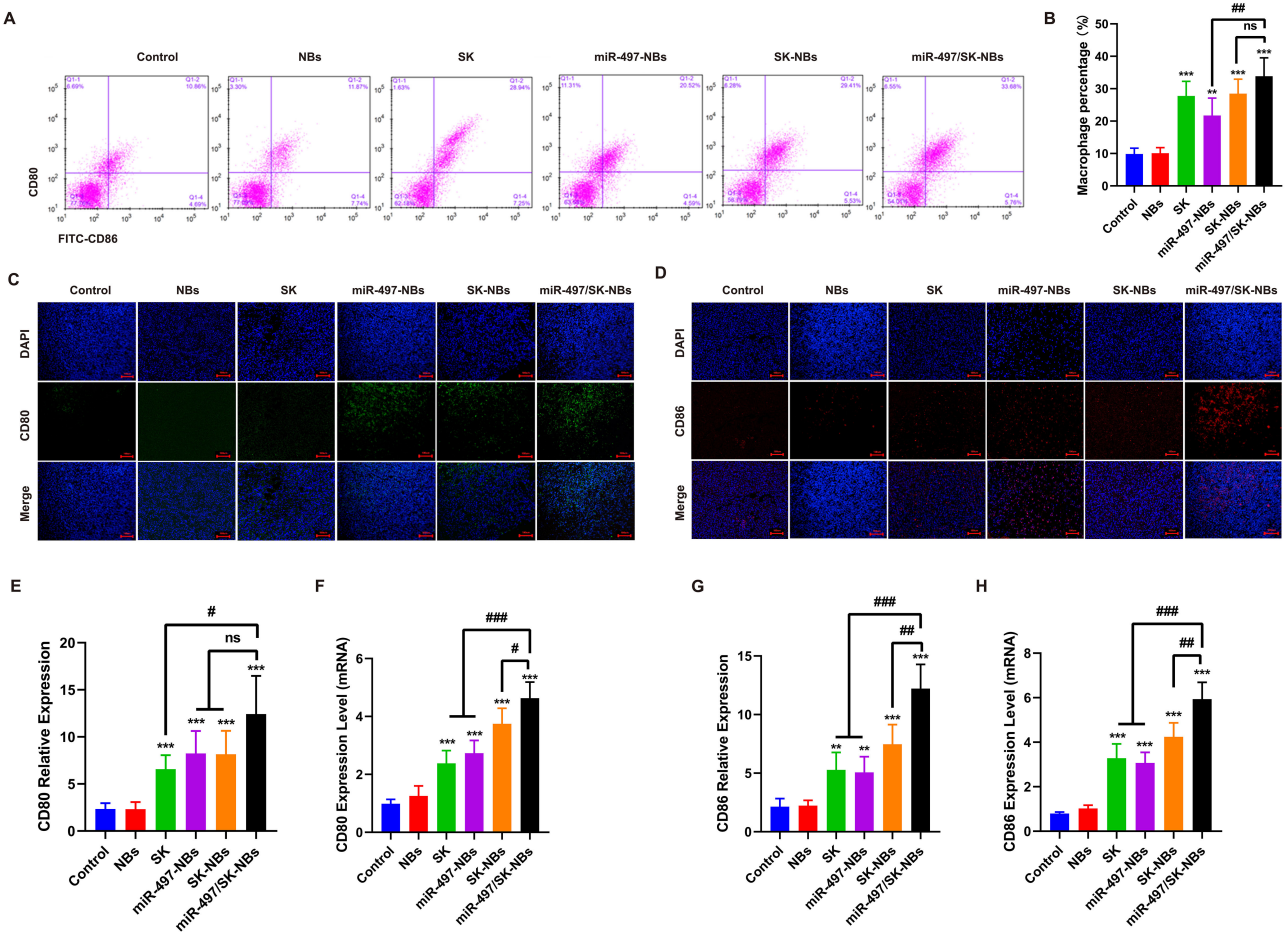
Analysis of macrophages cell activity by miR-497/SK-NBs *in vivo.***(A)** The ratio of CD80-/CD-86-labeled macrophages cells was detected by flow cytometry. **(B)** Quantitative analysis of macrophages cells percentage from panel **(A)**. **(C)** The expression of CD80 in tumor tissues was detected by immunofluorescence, (magnification, 200×), scale bar 100 μm. **(D)** The expression of CD86 in tumor tissues was detected by immunofluorescence, (magnification, 200×), scale bar 100 μm. **(E)** Quantitative analysis of CD80 expression from panel **(C)**. **(F)** Quantitative analysis of CD80 mRNA levels in tumor tissues. **(G)** Quantitative analysis of CD86 expression from panel **(D)**. **(H)** Quantitative analysis of CD86 mRNA levels in tumor tissues. (Data are expressed as mean ± SD (n = 5). Statistical significances were calculated via ANOVA. ^**^P < 0.01, ^***^P < 0.001 versus the control group, ^#^P < 0.05, ^##^P < 0.01, ^###^P < 0.001 versus the miR-497/SK-NBs group).

ICD enhances the expression of co-stimulatory molecules CD80 and CD86 on macrophages, essential for T-cell activation and adaptive immune response. We investigated macrophage activation in spleen and tumor tissue via CD80 and CD86 expression (**Figure 6**). In our study, we observed that miR-497/SK-NBs elicited the most pronounced enhancement in macrophage activation in spleen, as evidenced by a significant increase in the proportion of CD80^+^ CD86^+^ cells. Specifically, the proportions of CD80^+^ CD86^+^ macrophages in mice treated with miR-497-NBs, SK-NBs, and miR-497/SK-NBs were 21.734 ± 5.393%, 28.465 ± 4.481%, and 33.865 ± 5.653%, respectively (**Figures 6A, B**). Subsequently, fluorescence analysis and RT-PCR were employed to detect the expression of CD80^+^ and CD86^+^, respectively. The results indicated that, in comparison to the Control and NBs groups, the FITC-labeled CD80 and APC-labeled CD86 expressions were enhanced in the Free-SK, miR497-NBs, SK-NBs, and miR497/SK-NBs groups, with the most pronounced fluorescence labeling observed in the miR497/SK-NBs group (**Figures 6C, D**). Quantitative fluorescence analysis and RT-PCR demonstrated that, relative to the Control group, the expression levels of CD80 and CD86 were significantly elevated, with the highest expression observed in the miR497/SK-NBs group (**Figures 6E-H**). These results suggests that through inducing tumor cell ICD effect the miR-497/SK-NBs can enhance CD80^+^ CD86^+^ macrophage activation, which for more effective immune response against tumors *in vivo*, as compared with that associated with the SK-NBs or miR497-NBs.

### 3.6 The miR-497/SK-NBs mediated PD-L1 down-regulation and enhaned the tumoricidal efficacy of T cells

**Figure 7:**
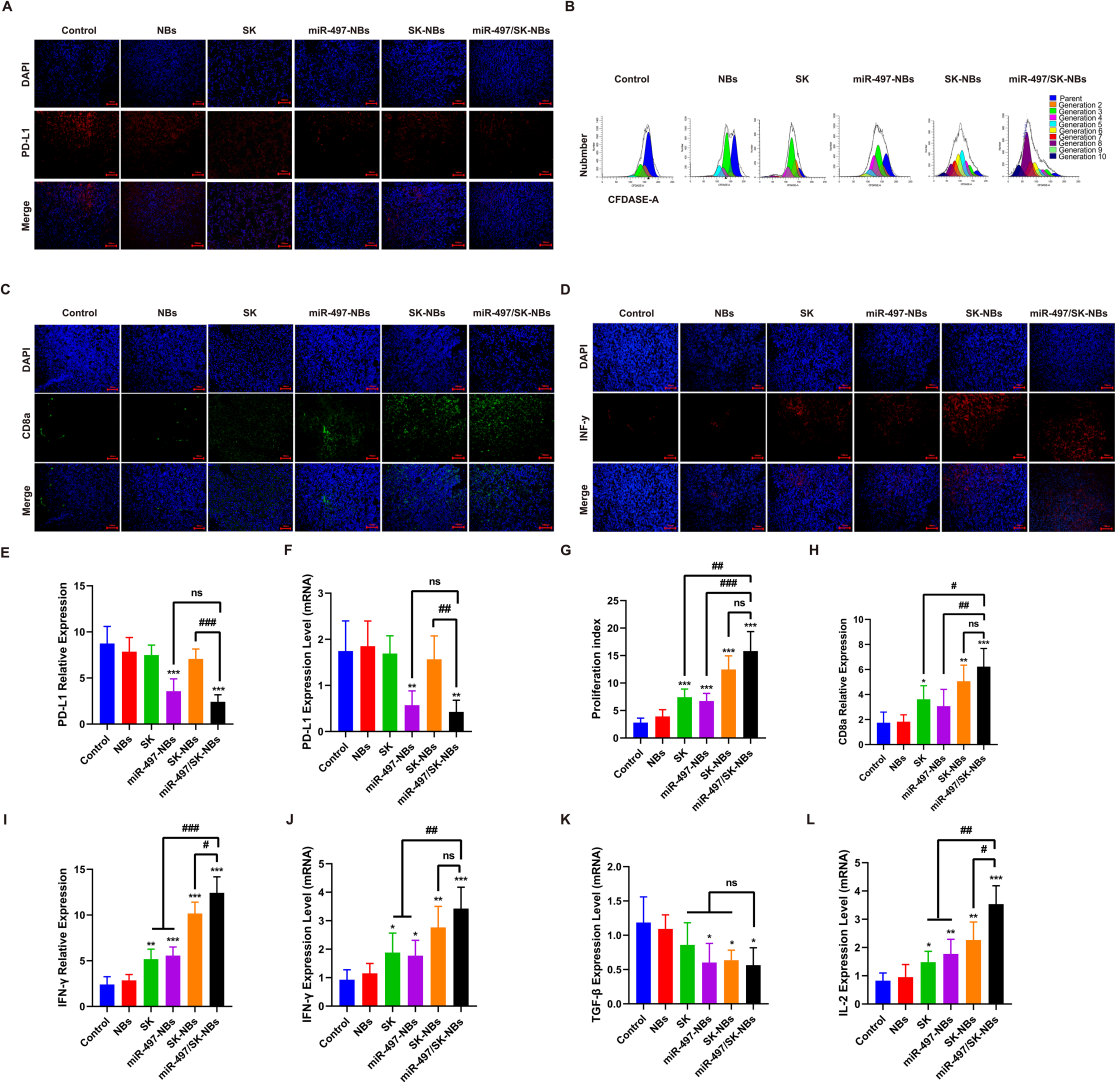
Investigation of CTLs activity and immune-related factors modulated by miR-497/SK-NBs *in vivo*. **(A)** The expression of PD-L1 on the surface of tumor tissues subjected to various treatments was examined using fluorescence microscopy, (magnification, 200×), scale bar 100 μm. **(B)** Lymphocyte proliferation in each experimental group was assessed via flow cytometry. **(C)** CD8a expression in tumor tissues was evaluated through immunofluorescence, (magnification, 200×), scale bar 100 μm. **(D)** IFN-γ expression in tumor tissues was similarly assessed by immunofluorescence, (magnification, 200×), scale bar 100 μm. **(E, F)** Quantitative analyses were conducted to determine PD-L1 protein and mRNA levels in tumor tissues. **(G)** Quantitative assessment of the lymphocyte proliferation index was performed from panel **(B)**. **(H)** Quantitative analysis of CD8a expression was derived from panel **(C)**. **(I, J)** Quantitative analyses of IFN-γ protein and mRNA levels in tumor tissues were conducted. **(K, L)** The expression levels of TGF-β mRNA and IL-2 mRNA were quantified. (Data are expressed as mean ± SD (n = 5). Statistical significances were calculated via ANOVA. ^*^P < 0.05, ^**^P < 0.01, ^***^P < 0.001 versus the control group, ^#^P < 0.05, ^##^P < 0.01, ^###^P < 0.001 versus the miR-497/SK-NBs group).

Given the substantial efficacy of miR-497/SK-NBs in delivering miR-497, we further investigated the potential for PD-L1 down-regulation following miR-497 delivery via NBs at the tumor site. PD-L1 protein and mRNA expression levels were quantified using immunofluorescence and qRT-PCR, respectively. The results indicated a significant reduction in PD-L1 protein expression mediated by miR-497-NBs (3.572 ± 1.339%) and miR-497/SK-NBs (2.425 ± 0.753%) compared to the control group (8.745 ± 1.854%) at the tumor site (**Figures 7A-E**). Additionally, qRT-PCR analysis revealed a corresponding decrease in PD-L1 mRNA levels, aligning with the observed reduction in PD-L1 protein expression, as depicted in (**Figures 7F**)

To explore the potential of miR-497/SK-NBs in enhancing anti-tumor immunity *in vivo*, we evaluated the immune responses of both splenic lymphocytes and tumor tissues. Initially, we performed an *in vitro* analysis of splenocyte proliferation. Splenocytes were labeled with CFDA-SE, and their proliferation was subsequently assessed using flow cytometry by measuring the CFDA-SE signal intensity (**Figure 7B**). The results indicated that lymphocyte proliferation in the miR-497/SK-NBs group (15.832 ± 3.531%) was approximately 5.6 times greater than in the control group (2.812 ± 0.823%) (**Figure 7G**). These findings suggest that the combined miR-497/SK-NBs therapy may enhance the proliferative capacity of lymphocytes in mice.

Reducing PD-L1 expression inhibits the PD-1/PD-L1 pathway, thereby boosting T cell activity in the tumor microenvironment. CD8a expression is a key biomarker for evaluating T cell activation and functionality, aiding in the assessment of immune responses in clinical settings. Consequently, we examined the expression of CD8a induced by the administration of drug-loaded NBs.

The study revealed that CD8a cell expression in the miR-497/SK-NB cohort was nearly threefold greater than in the control group (**Figures 7C-H**). The production of IFN-γ by CD8+ T cells, an indicator of antigen-specific immune responses, was evaluated using immunofluorescence analysis and RT-PCR. As depicted in **Figures 7D, I, J** treatment with miR-497/SK-NBs led to the highest count of CD8^+^ IFN-γ-producing cells in mice. Furthermore, to comprehensively assess the regulatory effects of the miR-497/SK-NBs combination therapy on *in vivo* anti-tumor immune activity, we measured the expression levels of immune-related mRNAs, including IL-2 (**Figure 7L**) and TGF-β (**Figure 7K**), across various tissues using RT-qPCR.The findings indicated that, relative to the control group, IL-2 levels increased by a factor of 3.5, while TGF-β levels decreased by a factor of 2.These results imply that the combined miR-497/SK-NBs therapy has the potential to significantly enhance anti-tumor immune responses.

## 4 Discussion

In our study, ultrasound-targeted nanobubble destruction (UTND) technique was used to enhance drug delivery and gene therapy. The principle behind UTND involves the use of ultrasound waves to induce cavitation in microbubbles, leading to their destruction. This destruction creates localized mechanical forces that can enhance the permeability of cell membranes, allowing for more efficient uptake of therapeutic agents, such as drugs or genetic material, into target cells (23). One of the significant advantages of UTND is its non-invasive nature, which allows for real-time monitoring of treatment efficacy through imaging techniques. What’s more, that UTND can remodel the tumor microenvironment, improving the efficacy of cancer immunotherapy by enhancing the infiltration of immune cells into tumors (24). So, we developed innovative nanobubble systems designed to deliver Shikonin and the gene miR-497. The study found that miR-497/SK-NBs had a particle size of about 258 nm, indicating strong miRNA binding and efficient Shikonin encapsulation. Then, we investigated the tumor inhibition efficiency of miR-497/SK-NBs. The antitumor effect *in vivo* was shown the miR-497/SK-NBs group exhibited the strongest tumor inhibition.

To avoid attack by the immune system, tumor cells also employ different strategies to evade immune surveillance such as low immunogenicity, which allows them to escape detection by immune cells (25). Consequently, it is crucial to modify the tumor’s low immunogenic microenvironment to achieve effective tumor immunotherapy. Prior research has demonstrated that ICD can activate the immune microenvironment within tumors, thereby enhancing the efficacy of immunotherapeutic interventionsADDIN. Pharmacological agents capable of inducing ICD in tumor cells are collectively termed ICD inducers. In this study, SK is identified as an ICD inducer that significantly impedes tumor cell proliferation, as evidenced by tumor growth curves. The findings presented in **Figure 5** indicate that SK induces ICD, as evidenced by the presence of key markers such as CRT and HMGB1. Furthermore, increased levels of CRT, HMGB1 and ATP in tumors following various NBs treatments were correlated with enhanced infiltration of CD80^+^ CD86^+^ macrophages and T cells. The study demonstrated that the ICD effect induced by SK *in vivo* was augmented by NBs and further synergistically enhanced when combined with miR-497 via the NBs delivery platform. This effect may be attributed to the upregulation of CRT and HMGB1, which potentially enhances tumor cell phagocytosis, stimulates the infiltration of effector immune cells, and synergizes with PD-L1 inhibition to effectively impede tumor progression.

ICD is vital for boosting the immune response to cancer by activating antigen-presenting cells (APCs), especially macrophages that express costimulatory molecules like CD80 and CD86 (26). These molecules play a significant role not only in T cell activation, but also influence the maturation of dendritic cells, which are pivotal for initiating and regulating immune responses (27). The expression of CD80 and CD86 on macrophages is closely associated with their antigen-presenting capabilities and the activation of T cells. In this study, we evaluated the levels of CD80 and CD86 in tumor tissues and observed an upregulation in the miR-497-NBs+US and miR-497/SK-NBs+US treatment groups. These findings suggest that tumor immunogenic cell death can activate CD80+ CD86+ macrophages, thereby enhancing the immune response against tumors.

The role of miR-497 in regulating programmed PD-L1 expression has garnered significant attention in recent cancer research. Reduced miR-497-5p levels are linked to higher PD-L1 expression in clear cell renal cell carcinoma. This highlights miR-497-5p as a potential therapeutic target to modulate PD-L1 levels and enhance the effectiveness of PD-1/PD-L1 immunotherapies (28). To investigate the target-effect relationship and elucidate the regulatory mechanism between miR-497 and PD-L1, we conducted experimental validation of the interaction between miR-497a and PD-L1, the results demonstrating that the transfection of miR-497a-5p significantly suppresses PD-L1 expression. In this experiment, NBs showed a well miRNA loading. Concurrently, when bound to NBs, it is protected from nuclease-mediated degradation in the bloodstream (29). Consequently, the conjugation of miR-497a with NBs emerges as a promising therapeutic strategy for cancer treatment.

PD-L1 down-regulation significantly impacts the tumor immune microenvironment, affecting tumor growth and immune response (30). PD-L1 is an essential immune checkpoint molecule that facilitates tumor evasion of immune surveillance. When PD-L1 expression is decreased, it can lead to a more favorable immune environment for anti-tumor responses, as evidenced by increased infiltration of cytotoxic T cells and enhanced immune activity against tumor cells (31). For instance, studies have shown that inhibiting PD-L1 can enhance the effectiveness of immune checkpoint inhibitors, leading to improved outcomes in various cancer models, including renal cell carcinoma and HCC (32). Thereby, we explored if miR-497-mediated PD-L1 downregulation could alter the tumor immune microenvironment and enhance immune-mediated tumor destruction.The findings indicated that miR-497 and SK on the surface of NBs enhance immune function by activating CD8^+^ T cells, releasing immune inflammatory factors, and promoting the ICD of tumor cells within the tumor microenvironment. The study revealed a significant increase in lymphocyte proliferation and CTL cell activation in the miR-497/SK-NBs+US group.The cancer cell death induced by blocking the PD-1/PD-L1 pathway has been shown to be intricately linked to the enhancement of CTL activity, particularly through the increased production of IFN-γ and various cytotoxic molecules (33). IFN-γ and IL-2 are essential in the immune response against cancer by enhancing the cytotoxic activity of immune cells such as T cells and NK cells (34). CD8a, part of the CD8 glycoprotein complex, is predominantly found on cytotoxic T cells, crucial for identifying and destroying cancer cells. Studies have demonstrated that CD8+ T cells, particularly those expressing CD8a, are pivotal in mediating anti-tumor immunity (35). As above cytokine secretions are critical component of the immune response, we detected different treatments to release cytokines, including IFN-γ, IL-2 and CD8a cytokines associated with cellular immunity. The levels of IFN-γ, CD8a and IL-2 were increased in the miR-497/SK-NBs+US group and the miR-497-NBs+US group. This suggests that therapeutic strategies aimed at not only enhance activating of CLT but also reshaping the immune factor infiltration within the tumor microenvironment, ultimately leading to improved therapeutic efficacy.

The experimental design seeks to co-deliver SK and miR-497a-5p via NBs to synergistically enhance the effectiveness of immunotherapy against liver cancer. Evaluations of tumor growth and apoptosis in tumor cells revealed that the group receiving the combined treatment exhibited the slowest tumor growth trajectory and the highest tumor inhibition rate. These results indicate that miR-497/SK-NBs exhibit the most significant efficacy in inhibiting liver cancer growth. Subsequently, the induction of ICD was evaluated across each treatment group. The results demonstrated that the miR-497/SK-NBs complex markedly upregulated the CRT, HMGB1 and ATP, thereby promoting the activation of CD80^+^ CD86^+^ macrophages. Furthermore, the miR-497/SK-NBs treatment group significantly augmented tumor tissue necrosis, liquefaction, and apoptotic body formation by facilitating tumor cell apoptosis. The mechanism involved increased expression of the pro-apoptotic factor Bax and decreased expression of the anti-apoptotic factor Bcl-2. Within the context of PD-L1 downregulation on tumor cells mediated by miR-495, the group receiving the combined treatment exhibited enhanced proliferation of splenic lymphocytes, an increased proportion of macrophages in the splenic suspension, and augmented T cell activation. Additionally, the expression levels of immunostimulatory factors, including CD8a, IFN-γ, and IL-2, were upregulated, whereas the immunosuppressive factor TGF-β was downregulated. These findings suggest that miR-497/SK-NBs, when mediated by ultrasound, induce a more robust anti-tumor immune response, thereby offering novel opportunities for enhancing immunotherapeutic strategies in HCC.

Despite the encouraging results observed in mouse models, significant challenges remain before this therapy can be applied to humans. Firstly, there are substantial immunological and pharmacokinetic differences between animal models and humans. Secondly, due to the complex biodistribution of nanocarriers, potential off-target effects remain a significant concern: non-specific accumulation could lead to abnormal miR-497 regulation of the cell cycle in healthy tissues, triggering unforeseen side effects. Furthermore, the long-term safety profile of this system remains difficult to predict. For example, the prolonged retention of nanobubble materials and their degradation products within the body may induce chronic inflammation or organ toxicity, while their potential to trigger immunogenic responses necessitates a systematic evaluation. Lastly, at the scale-up manufacturing level, transitioning from laboratory preparation to large-scale, highly reproducible production meeting clinical demands presents a major bottleneck. Ensuring consistent nanoparticle size, drug loading capacity, and release kinetics across batches is a prerequisite for industrialization.

## 5 Conclusion

In conclusion, the multifunctional miR-497/SK-NBs developed in this research not only induced ICD through Shikonin but also facilitated the transition of tumors from a state of low immune responsiveness to one characterized by enhanced immune activity. Furthermore, in conjunction with miR-497, the multifunctional NBs elicited a robust and sustained systemic immune response by down-regulating PD-L1. The proposed combinatorial strategy shows considerable promise for application across a diverse array of solid tumors, which exhibit limited responsiveness to ICB therapy. This approach presents exceptional potential for future advancements in the field.

## Data Availability

The datasets presented in this study can be found in online repositories. The names of the repository/repositories and accession number(s) can be found in the article/supplementary material.
